# Curing cancer? Further along the new pH-centric road and paradigm

**DOI:** 10.18632/oncoscience.422

**Published:** 2018-06-24

**Authors:** S. Harguindey, T. Koltai, S. J. Reshkin

**Affiliations:** Salvador Harguindey, Institute of Clinical Biology and Metabolism, 01004 Vitoria, Spain

**Keywords:** pH paradigm in cancer, cancer treatment

During the last few years, the understanding of the dysregulated hydrogen ion dynamics and reversed proton gradient of cancer cells has resulted in a new and integral pH-centric paradigm in oncology, a model which embraces most if not all aspects of cancer, from etiopathogenesis to treatment. The cancer-selective abnormality of intracellular alkalinization along with extracellular acidification (“proton gradient reversal”) of all types of solid tumors and leukemic cells is finally recognized as a specific and most selective hallmark of malignancy. As a consequence of this acid-base homeostatic failure of cellular hydrogen ion (H+) dynamics, the attempt to induce cellular acidification using proton transport and pump inhibitors (PTIs) and other intracellular acidifiers of different origins is becoming a new therapeutic concept and selective target of cancer treatment. A full issue containing fourteen reviews on the different aspects of the new pH-centric anticancer paradigm has been recently published [1].

## A “final” cause for breast cancer?

Recently, it has been demonstrated that H+ efflux alone is sufficient to induce dysplasia and potentiate growth and invasion by oncogenic Ras and, furthermore, that inhibiting this H+ efflux produced cell death in invasive primary tumor cell lines through intracellular acidification and NHE inhibition. Similar results have been obtained by Fliegel`s group, showing that NHE-mediated H+ extrusion by itself has a carcinogenic effect on breast cells. In these studies, NHE1 hyperactivity appears to be an early and decisive driver in breast cancer carcinogenesis. Furthermore, an elevated pHi and increased proton efflux with a secondary acidified microenvironment (PCR) has been implicated in the transition and progression from precancerous ductal carcinoma *in situ* to invasive breast cancer, with the precancerous lesion already showing a higher than normal proton export rate [2]. We have to agree with these authors that H+ efflux and/or intracellular alkalinization, previously induced by a wide array other etiological factors of many different natures appear to be the final mediating cause of cancer, at least of triple-negative breast cancer. It has been finally concluded that NHE-1 inhibitors could play both a preventive role in breast cancer pathogenesis and also in its treatment [2, 3].

## A universal mediating mechanism as the integral and “final” cause of all cancers?

Recently, we integrated a large myriad of known carcinogenic factors from many different natures known to be the main cause of cancer via a universal mediating mechanism: an increase of cell pH and/or stimulation of NHE activity (see Table 2 in reference) [3]. However, pH homeostasis is such an important issue for cell transformation, growth and survival that, in addition to NHE overexpression, the proton extrusion system is also mediated by a cohort of other membrane-bound proton pumps transporters and ion channels. In this line, other important participants in elevating pHi and/or preventing cellular acidification and low pH-mediated apoptosis are carbonic anhydrase IX and XII, vacuolar H+-ATPase proton pumps, voltage gated sodium channels, sodium bicarbonate cotransporters and monocarboxylate transporters (MCTs) [4].

## On the new therapeutic road

Beyond cariporide [5], among the new and potent NHE1 inhibitors, the so-called “Compound 9t” could very well become a “magic bullet” drug for a number of human malignancies. This is because Compound 9t (a 5-aryl-4-(4-(5-methyl-1H-imidazol-4-yl) piperididn-1-yl) pyrimidine analog), has been shown to be 500-fold more potent against NHE1 than Cariporide [5] and to have a greater selectivity for NHE1 over NHE2 (1400-fold). Additionally, compound 9t is orally bioavailable, has low side effects in mice and shows a significantly improved safety profile over other NHE1 inhibitors. Surprisingly, Compound 9t has never been tested as an anticancer drug to date in spite of its most promising antitumoral characteristics and selective anticancer potential [6]. The concerted utilization of carbonic anhydrase, proton pumps and voltage gated sodium channels inhibitors, together with the new, potent and selective NHE-1 inhibitors may create a very hostile environment for cell proliferation, tumor growth, invasion and metastatic spread [3, 4, 6].

## CONCLUSIONS

Cancer research has always been about finding the weak points of malignant tumors in order to target them selectively and avoid damaging normal cells. This “magic bullet-like” approach to treatment demands knowing the main selective characteristics of cancer cells and tissues that are not present in normal cells. The pH dynamics of cancer cells, and specifically, the pH- or proton gradient reversal, now appears as a specific abnormality and even the most differential metabolic trait of all and each type of cancer. Therefore, this new cancer paradigm should and hopefully soon would be successfully exploited in any approach to selective cancer therapeutics [3, 7].
